# Day Programs for children and adolescents with eating disorders: A systematic review

**DOI:** 10.1002/erv.2953

**Published:** 2022-11-09

**Authors:** Govind Krishnamoorthy, So Min Shin, Bronwyn Rees

**Affiliations:** ^1^ School of Psychology and Wellbeing Centre for Health Research University of Southern Queensland Ipswich Queensland Australia; ^2^ Young Minds Psychology Ipswich Queensland Australia

**Keywords:** adolescent health, eating disorders, day programs, partial hospitalization

## Abstract

Day programs have received significant consideration within psychological literature as part of a continuum of mental health services. With increasing attention on the prevalence of eating disorders in children and adolescents, and the need for early intervention to minimize the costs and burden of the disorder, eating disorder day programs (also referred as partial hospitalization) have begun to emerge around the world. Despite their widespread use, no reviews to date have examined the efficacy of day programs for the treatment of eating disorders in children and adolescents. The current narrative literature review aims to describe and evaluate the efficacy of day programs for children and adolescents. The literature review was conducted according to the PRISMA guidelines and aimed to explore the outcomes and common program elements of day programs to guide clinical practice and service development. The review found variations amongst the day programs related to program elements, measures utilized and outcomes. Overall, the results suggest that day programs for children and adolescents are effective at restoring body weight, reducing eating disorder symptoms and addressing comorbid mental health concerns.

## Key points

- Day programs (DP)for children and adolescents with eating disorders are becoming increasingly prevalent around the world. These programs vary in their structure, processes and efficacy
- The systematic review of 21 programs revealed day programs as effective in restoring body weight, reducing eating disorder symptoms and addressing comorbid mental health concerns. There is evidence to suggest the maintenance of these gains at 3 and 6 month follow‐up
- The review highlights promising models of care, and provides guidance on evidence‐based clinical processes, as well as program evaluations of day programs

**Abbreviations**
ABAS: adaptive behaviour assessment system
ADHD: attention deficit hyperactivity disorder
AN: anorexia nervosa
ARFID: avoidant and restrictive food intake disorder
BMI: body mass index
BN: bulimia nervosa
CBT‐E: cognitive behaviour therapy – enhanced
CDI: children’s depression inventory
ChEAT: children’s eating attitudes test
DBT: dialectical behaviour therapy
DP: fay programs
DSM: diagnostic and statistical manual
EBW: expected body weight
ED: eating disorders
EDE‐Q: eating disorder examination questionnaire
EDI: eating disorder inventory
EPHPP: effective public health practice project
FBT: family based treatment
HLC: higher levels of care
IBW: ideal body weight
MASC: Multidimensional Anxiety Scale for Children
MDT: multidisciplinary team
MRAOS: Morgan and Russel average outcome score
OSFED: other specified feeding and eating disorder
PvA: parents versus anorexia scale
RCMAS: revised children’s manifest anxiety scale
USFED: unspecified feeding and eating disorder

## 1 INTRODUCTION

Eating disorders (EDs) are complex, prevalent yet treatable mental illnesses, with a high rate of onset in adolescence (Lawrence et al., 2015). EDs in children and adolescents are particularly harmful as they interfere with normal growth and development (American Psychiatric Association, 2013) and have one of the highest mortality rates of all psychological disorders (Chesney et al., 2014). This is in part due to medical complexities associated with EDs, for example Anorexia Nervosa (AN), an ED involving the restriction of food intake resulting in life‐threatening medical issues associated with significant weight loss (APA, 2013). Psychological treatment for children and adolescents with EDs typically involves community‐based, outpatient services (Derenne, 2019). For children experiencing severe and chronic ED symptoms, mental health services offering more intensive forms of treatment, referred to as Higher Levels of Care (HLC), are often recommended (Derenne, 2019). HLC represents a continuum of services in hospital based inpatient units or residential settings. These services typically serve children and adolescents who are medically compromised due to EDs or have not made sufficient progress in an outpatient clinic and require the child and their family to stay at the facility. These treatment settings aim to match illness severity with treatment dosage – such as increased medical monitoring, supervision of meals, the provision of multidisciplinary treatment programs and increased frequency of psychological treatment sessions (Anderson et al., 2019; Derenne, 2019).

Day Programs (DPs) (also referred to as partial hospitalisation) is useful for those whose symptoms are too severe to be treated by a traditional outpatient team, but who do not require hospital based, inpatient or a residential treatment (Derenne, 2019). At DPs, clients typically receive 6–10 hours per day of treatment and supervised meals for 3.5–5 days per week (Simic et al., 2018). DPs have been found to facilitate increased family involvement in treatment and to be cost‐effective given the high costs associated with inpatient and long‐term residential services for clients with severe and refractory forms of EDs (Derenne, 2019). Although DPs are widely used to treat EDs, no reviews have focused on examining the structure and effectiveness of DPs in treating EDs in child and adolescent populations. Previous reviews have focused on the use of DP for both adult and adolescent populations – with minimal discussion of the utility and efficacy of these services for children (e.g., Friedman et al., 2016). Hence, it is crucial to evaluate the effectiveness of DPs and examine their program contents to guide clinical practice for children and adolescents with EDs. This narrative literature review will aim to provide a useful guide for researchers and clinicians planning to establish a program. The review will aim to address the following two questions:

1. Are DPs for children and adolescents efficacious in restoring psychological and physical health?
2. What are the common program elements of DPs for children and adolescents with EDs?

## 2 METHOD

### 2.1 Search strategy

A review of peer‐reviewed literature was performed by the authors between January 2020 to November 2021 to evaluate the effectiveness of Day Programs (DP) in improving physical (e.g., weight) and psychological health (e.g., ED related symptoms, depression, anxiety) among children and adolescents. In addition to health outcomes, the narrative literature review aimed to identify common program elements of DPs. Given the focus on understanding the efficacy of ED‐DPs, only peer‐reviewed studies were included, and grey literature was not accessed for the review. When further information regarding services and programs were required, the authors directly contacted the authors of reviewed studies requesting further details. The review was performed using the following six databases: PsycInfo, PsycArticles, PubMed, Ebscohost Megafile Ultimate, CINAHL and Psychology and Behavioural Sciences. The search strings utilized are displayed in Table 1. The reference lists of the included studies were also reviewed to further identify suitable studies that met the inclusion criteria.

**TABLE 1:** Search terms

Key concepts	String 1	String 2	String 3
	Eating disorder	Population	Service Delivery
Search Terms	anorexia nervosa OR bulimia nervosa OR eating disorder not otherwise specified OR other specified feeding and eating disorder OR avoidant restrictive food intake disorder OR eating disorder*	adolescen* OR child* OR youth	day program OR day hospital* OR partial hospital*

### 2.2 Selection criteria

Included studies met the following criteria:

1) Studies published between 2009 and 2021
2) Inclusion of participants who were diagnosed with any type of EDs using DSM‐IV, DSM‐IV‐TR, or DSM‐5 criteria
3) Participants were aged 18 years or younger.
4) Collection of quantitative outcomes which must include a measure of body weight

Excluded studies met the following criteria:

1) All participants of the research were aged 19 years and above.
2) Studies that did not assess body weight (e.g., Body Mass Index (BMI), Ideal Body Weight (IBW))
3) Studies not written in English
4) Studies that were not available to view in full text
5) Studies that were not peer‐reviewed

**FIGURE 1:**
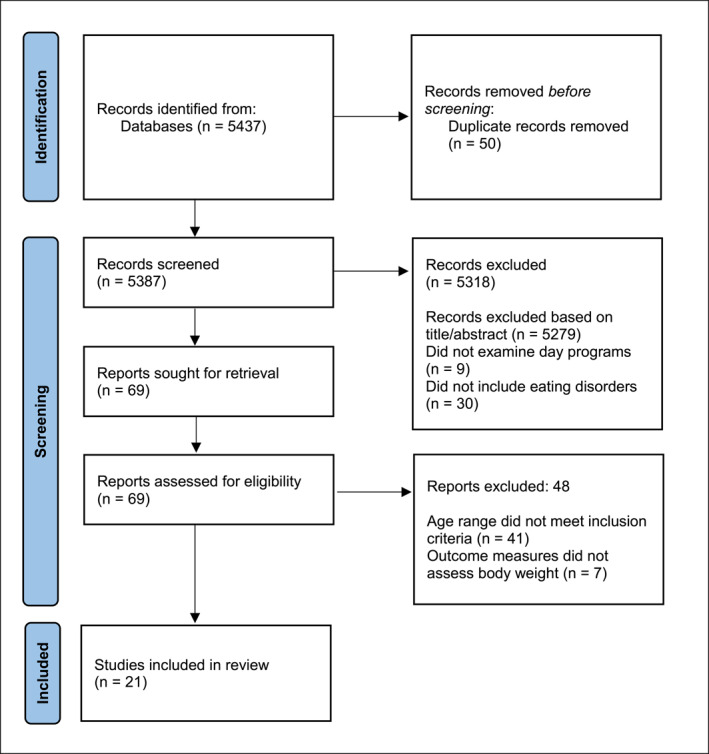
PRISMA flowchart of the systematic literature review.

The initial database search identified a total of 5437 articles and 50 duplicate articles were removed. Titles and abstracts of the articles were individually and manually screened in reference to the inclusion and exclusion criteria, leaving 71 articles. A manual examination of the full text was conducted in a similar manner, further removing 50 articles from the analysis. The full text of the remaining 21 articles were read to ensure that they met the eligibility requirements (see Figure 1 below).

### 2.3 Data extraction and management

The Effective Public Health Practice Project (EPHPP) Quality Assessment Tool (Thomas et al., 2003) was used to evaluate the methodological quality of each article. Using the EPHPP, nine areas of selection bias, study design, blinding, data collection methods, withdrawals and drop‐outs for discharge and follow‐up data, analysis, and data collection for discharge and follow‐up data were assessed. The quality in the overall design of the 21 articles were appraised by both the research student and the supervisor. All 21 studies were selected for inclusion.

### 2.4 Results

All studies collected quantitative data and employed a longitudinal study design. One third of studies included follow‐up data post‐discharge (*n* = 7). One article compared patient outcomes between different levels of care (i.e., DP vs. inpatient). The following section will present the narrative summary of the ED‐DP services and service outcomes.

#### 2.4.1 Participants

The 21 studies included a total of 1366 participants, averaging 65 participants per study with the range varying from *n* = 19 (Girz et al., 2013) to *n* = 160 (Lazaro et al., 2011). Most participants were female, with eight studies including only female participants (Bryson et al., 2018; Goldstein et al., 2011; Griz et al., 2013; Henderson et al., 2014; Herpertz‐Dahlmann et al., 2014; Lazaro et al., 2011; Zanna et al., 2021).

#### 2.4.2 Age

All participants from the included studies had participants between the ages of 7–18 years. However, participants from two studies included participants older than 18 years, not meeting the age criteria (deGraft‐Johnson et al., 2013; Hoste, 2015). Despite the violation of the inclusion criteria for age, they were included as most of the participants were aged under 18 years old (e.g., deGraft‐Johnson et al.’s (2015) study included 174 participants aged less than 18 years – out of a total of 198 patients).

#### 2.4.3 Eating disorder diagnoses

ED diagnoses were determined using the DSM‐5 in six studies (Hoste, 2015; Ornstein et al., 2017; Bryson et al., 2018; Pennell et al., 2019; Simic et al., 2018; Smith et al., 2021), and using DSM‐IV or DSM IV‐TR in the remaining fifteen studies. Four studies included participants with AN only (Herpertz‐Dahlmann et al., 2014; Ngo & Isserlin, 2014; Martin‐Wagar et al., 2019; Rienecke, 2019) while the other studies included participants with various EDs such as AN, BN, EDNOS, ARFID, OSFED or UFED.

#### 2.4.4 Comorbidities

Ten studies reported on comorbid psychiatric disorders (Bustin et al., 2013; Rienecke, 2019; Ornstein et al., 2012; Girz et al., 2013; Grewal et al., 2014; Herpertz‐Dahlmann et al., 2014; Bryson et al., 2018; Simic et al., 2018; Pennell et al., 2019; Martin‐Wagar et al., 2019). Mood disorders was the most common comorbid diagnosis followed by Anxiety Disorders and Attention Deficit Hyperactivity Disorder (ADHD).

#### 2.4.5 Sites

The countries in which the ED‐DPs were located were: the United States of America (*n* = 9), Canada (*n* = 5), Australia (*n* =1), Spain (n =1), Germany (*n* =1), United Kingdom (*n* = 2) and Italy (n = 2). Several DPs were located on the grounds of pediatric hospitals (*n* = 10), general psychiatric hospitals (*n* = 2), and general hospitals (*n* = 8; Table 2).

**TABLE 2:** Tabulation of articles included in the review

Study and Program Details	Study Design	Participant Characteristics	Measures Used	Key Outcomes	Limitations
Baudinet et al., 2020 **Program**: Intensive Day Treatment Program, Maudsley Hospital, London. **Country**: United Kingdom	Case seriesx	**N** = 130 **Diagnostic groups**: AN = 93.84% EDNOS = 6.16% **Gender**: Female = 94.61% Male = 5.49% **Age range**: 11–18 years *M* = 15.02 *SD* = 1.52	**Weight Measure**: Percentage of Body Mass Index (% *m*BMI) adjusted for age and gender **Mental Health Measures**: Modified Morgan‐Russel Global Outcome Assessment Schedule (MRGOAS) Eating Disorder Inventory 3rd Edition (EDI‐3; Garner, 2004) EDI‐3 Mood and Feelings Questionnaire (MFQ) Development and Wellbeing Assessment (DAWBA) Schedule of Non‐adaptive and Adaptive Personality for Youth (SNAPY‐Y) Five Factor Obsessive Compulsive Inventory ‐ Short form (FFOCI‐SF) Temporal Experience of Pleasures ‐ Anticipatory and Consummatory (TEPS‐ANT; TEPS‐CON) Emotion Regulation Questionnaire (ERQ) Withdrawal Subscale of the Youth Self‐Report questionnaire (WS‐YSR) Social Connectedness Scale (SCS‐R) Attachment Styles Questionnaire (ASQ)	**Physical Health Outcomes**: Over 13.40 weeks, patients showed weight improvement Mean gain of 7.18% *m*BMI. Pre *m*BMI = 82.39 (846), Post *m*BMI = 89.51 (8.59) **Mental Health Outcomes:** MRGOAS scores across three ranges at discharge: MRGOAS‐Good = 53.08%, N = 69; MRGOAS‐Intermediate = 17.69%, N = 23 MRGOAS‐Poor = 28.46%, N = 37. Reduction in eating disorder symptomology in EDI‐3: Drive for thinness *p* < 0.01; *d* = 0.33 Low self‐esteem *p* < 0.05; *d* = 0.26 Personal alienation *p* < 0.01; *d* = 0.39 Interpersonal insecurity *p* < 0.05; *d* = 0.26 Interceptive deficits *p* = 004; *d* = 0.38 Ascetism *p* < 0.05; *d* = 0.29 Improvement in depression symptoms, as measured by MFQ. *p* < .01; *d* = 0.41 Reduction in inflexibility, as measured by FFOCI‐SF *p* < 0.01; *d* = 0.34 Increases in anticipatory and consummatory experiences of pleasure, as measured by TEPS. Anticipatory (TEPS‐ANT) *p* < 0.001; *d* = 0.42 Consummatory (TEPS‐CON) *p* < 0.001; *d* = 0.51 Reduction in expressive suppression, as measured by ERQ. *p* < 0.001; *d* = 0.97 **Service Utilization Outcomes**: During or after treatment, 6 people were admitted to inpatient care.	As with all other similar studies, this study also relied on self‐reports to measure eating disorder symptomology and mood. The study adopted a shorter version of RO‐DBT (i.e., 12.40 weeks rather than the original 30). Patients may have had less time to consolidate DBT skills. Use of some measures that were not validated using adolescent sample (e.g., FFOCI). Lack of follow‐up data.
Bryson et al. (2018) **Program**: Partial Hospital Program at Penn State Health Children’s Hospital, Pennsylvania **Country**: United States of America	Case series	**N** = 62 **Diagnostic groups**: ARFID = 2.4%. AN = 97.6% **Gender**: All females. **Age range**: 7–17 years *M* (ARFID) = 11.43 yrs *SD* = 1.55 *M* (AN) = 14.12 yrs *SD* = 1.48	**Weight Measure**: Median Body Mass Index (MBMI) **Mental Health Measures**: Children’s Eating Attitudes Test (ChEAT; Maloney, McGuire, & Daniels, 1988)	**Physical Health Outcomes**: Improvement in MBMI between pre‐post *p* < 0.001, *r* = 0.88 Weight was maintained from post to follow‐up (31 months). Weight at follow‐up was slightly higher than discharge *p* = 0.18 AN group reported greater increase in %MBMI from intake to discharge *p* < 0.01, *r* = 0.37 **Mental Health Outcomes**: Reduction in Total scores on ChEAT from intake to discharge *p* < 0.001, *r* = 0.66 Reduction in Total scores on ChEAT from discharge to follow‐up *p* < 0.01, *r* = 0.34	Limited program descriptions regarding family involvement Limited description about multidisciplinary team approach. Low response rate (45.3%) for follow‐up data.
Bustin et al. (2013) **Program**: Partial Hospital Program at Pennsylvania State Hershey Children's Hospital, Pennsylvania. **Country**: United States of America	Case series	**N** = 30 **Diagnostic groups**: AN = 33% BN = 7% EDNOS = 60% **Gender**: Female = 86.7% Male = 13.3% **Age range**: 7–17 years *M* = 12.8 *SD* = 2.0	**Weight Measure**: Ideal Body Weight (IBW) **Mental Health Measures**: ChEAT Revised Children’s Manifest Anxiety Scale (RCMAS; Reynolds & Richmond, 1985). Children’s Depression Inventory (CDI; Kovacs, 1992). Motivational Stage of Change for Adolescents Recovering from an Eating Disorder (MSCARED; Bustin et al., 2013).	**Physical Health Outcomes**: Improvement in % IBW between admission and discharge. *p* < 0.0001; from 86% to 96%. **Mental Health Outcomes**: Reduction on ChEAT scores *p* < 0.001; from 24.7 to 11.8 Gains in motivation for change as measured by MSCARED, on both adolescent self‐report and parent report. *p* < 0.001	Retrospective cohort study, with no control group. Limited program description and multidisciplinary team approach. Small sample size. Limited reporting of effect sizes of change in body weight. Limited follow‐up data.
deGraft‐Johnson et al., 2013 **Program**: Day Program, Eating Disorder Center, Steven and Alexandra Cohen Children’s Medical Center of New York, New York. **Country:** United States of America	Case series	**N** = 198 <12 years = 24. 13–15 years = 73. 16–18 years = 77. >18 years = 24. **Diagnostic groups**: AN = 52.5% BN = 8.1% EDNOS = 39.4% **Gender**: Female = 96% Male = 4% **Age range**: *M* = 17.7 *SD* = 1.03	**Weight Measure**: Body weight (kgs)BMI	**Physical Health Outcomes**: Mean weight gain of 0.95 kgs from admission to end of treatment. Greater weight gain was positively correlated with diagnoses of AN or EDNOS (as compared to BN) *p* < 0.05	Missing the report on age range Unable to calculate mean age and the number of diagnoses for those aged 18 years or younger. Short length of stay (*M* =2.6 weeks) No inferential analysis of change in weight gain from admission to discharge. No measures of mental health. Missing follow‐up data or control group.
Girz et al., 2013 **Program**: Eating Disorder Program, Hospital for Sick Children, Toronto, Ontario **Country**: Canada	Case series	**N** = 19**Diagnostic groups**: AN‐R = 24% BN‐P = 35% EDNOS‐R = 35% EDNOS‐B/P = 6% **Gender**: All female **Age range**: 13‐18 yrs *M* = 16.06 *SD* = 1.03	**Weight Measure**: IBW **Mental Health Measures**: EDI‐3 Multidimensional anxiety scale for children (MASC; March, 1997)CDI Parents versus anorexia scale (PvA; Rhodes et al., 2005)Eating disorder Symptom Impact Scale (EDSIS; Sepulveda et al., 2008)	**Physical Health Outcomes**: Weight gain observed at 3‐month follow‐up: 63% of sample at IBW 37% % of sample at 94% IBW Weight gain observed at 6‐month follow‐up: 84% of sample at IBW 16% of sample at 99% IBW **Mental Health Outcomes**: Reduction in eating disorder symptoms on all subscales of EDI‐3, except maturity fears, at 3 months and 6 months *p* < 0.003, *Mean t‐score range across sample groups*: elevated = 67–99; typical = 25‐66; low = 1‐24 Reduction in depressive symptoms, as measured by CDI, at 3 months and 6 months. *p* < 0.005, *Average t‐score* = 45‐55 Reduction in anxiety symptoms in several sub‐scales of MASC at 3 months and 6 months. *p* < 0.02, *Average t‐score* = 45–55 **Family Functioning Outcomes**: Increase in parent self‐efficacy found on PvA at 3 and 6 months *p* < 0.02, *Average t‐score not reported.* Decrease in impact of eating disorders on family, as measured by EDSIS, at 3 months and 6 months *p* < 0.001, *Average t‐score not reported.*	Missing reports of program description on operating hours, dropout rates. Small sample size. Although all patients achieved 99 to 100% of IBW at the end of treatment, more than half of patients did not have problems with restrictive food intake and were not underweight in the beginning of treatment. No inferential statistics on changes in weight gain. Absence of control group
Goldstein et al., 2011 **Program**: Transition Program at Sydney Children's Hospital, Sydney, New South Wales **Country**: Australia	Case series	**N** = 28 **Diagnostic groups**: AN = 78.6% EDNOS‐R = 21.4% **Gender**: All female **Age range**: 12–18 years *M* = N/A	**Weight Measure**: IBW Body Mass Index (BMI) **Mental Health Measures**: Eating Disorders Inventory‐2 and 3 (EDI‐2; EDI‐3; Garner, 1991, 2004) The Eating Attitudes Test‐26 (EAT‐26; Garner et. al., 1982) The Anorexia Stages of Change Questionnaire (ANSOCQ; Rieger et al., 2000)	**Physical Health Outcomes**: Weight gain between pre‐treatment and post‐treatment, and six‐month follow‐up, as measured by BMI Pre‐post*: p* < 0.05; *d* = 0.35 Six‐month follow‐up: *p* < 0.01; *d* = 0.86 Weight gain between pre‐treatment and post‐treatment as measured by IBW Pre‐post*: p <* 0.05*; d =* 0.29 Six‐month follow‐up*: p <* 0.01*; d =* 0.70 **Mental Health Outcomes:** Reduction in eating disorder symptomology, as measured by EDI‐3 subscales. Drive for thinness Pre‐post: *p* < 0.01; *d* = 0.43 Six‐month follow‐up: *p* < 0.05; *d* = 0.99 Body dissatisfaction Six‐month follow‐up: *p* < 0.01; *d* = 0.84 Perfectionism Pre‐post: *p* < 0.05; *d* = 0.11 Six‐month follow‐up: *p* < 0.05; *d* = 0.49 Reduction in eating disorder symptomology, as measured by EAT‐26. Pre‐post: *p* < 0.01; *d* = 0.30 Six‐month follow‐up: *p* < 0.01; *d* = 0.78 Improvements in eating disorder symptomology and readiness for recovery, as measured by ANSOCQ. Pre‐post: *p* < 0.01; *d* = 0.58 Six‐month follow‐up: *p* < 0.05; *d* = 0.81.	10 patients were re‐admitted to IP while attending DP. Of these, 8 patients' data were included in the study. This makes it unclear whether improvement at post‐treatment was due to DP or IP or both. Small effect sizes on all measures Limited generalizability due to including patients with mild to moderate ED symptoms Absence of control group.
Grewal et al., 2014 **Program**: Eating Disorders Day Hospital program at The Hospital for Sick Children (SickKids), Toronto, Ontario. **Country**: Canada	Case series	**N** = 65 **Diagnostic groups**: AN‐R = 60% AN‐BP = 13.8% BN‐P = 7.7% BN‐NP = 3/1% EDNOS‐R = 6.2% EDNOS‐B/P = 6% BE = 3.1% **Gender**: Female = 94% Male = 6% **Age range**: 13–18 years *M* = 15.60 *SD* = 1.40	**Weight Measure**: Goal weight (GW) **Service Utilization Measure:** Length of stay in the program (days)	**Physical Health Outcomes**: 58.5% of patients who completed the program met their 100% GW and maintained GW at six‐month follow‐up. Patients who completed program had higher GW at discharge, compared to non‐completers. *p* < 0.0001; *t* = ‒4.33	Calculated GW formula not clearly described. Stringent criteria to remain in the program – based on early weight gain. Absence of control and post‐treatment outcomes.
Henderson et al., 2014 **Program**: Eating Disorder Day Treatment Program (EDDTP) **Country**: Canada	Case series	**N** = 65 **Diagnostic groups:** AN = 63.7% BN = 10.2% EDNOS = 26.1% **Gender**: All female **Age range**: 11–18 years *M* = 15.00 *SD* = 1.34	**Weight Measure**: BMI **Mental Health Measures**: EDI‐3 CDI MASC	**Physical Health Outcomes**: Weight gain between pre‐treatment and post‐treatment, and six‐month follow‐up, as measured by BMI. Pre‐post*: p* < 0.001; RCI = 42 (64.6% improved); Total in healthy weight range = 86.9% Six‐month follow‐up: *p* < .001; RCI = 8 (12.3% improved); Total in healthy weight range = 64.8% **Mental Health Outcomes:** Reduction in eating disorder symptomology, as measured by EDI‐3 subscales. Drive for thinness Pre‐post: *p* < .01; RCI = 26 (40% improved); Total in normal range = 29.5%. Pre‐Six‐month follow‐up: *p* < 0.001; RCI = N/A. Body dissatisfaction Pre‐Six‐month follow‐up: *p* < 0.01; RCI = N/A. Reduction in depressive symptoms, as measured by CDI. Pre‐post: *p* < .005; RCI = 26 (40%); RCI = 26 (40%); Total in normal range = 64.2%. Six‐month follow‐up: *p* < 0.01; RCI = 12 (18.5%); Total in normal range = 53.3%. Reduction in anxiety symptoms, as measured by MASC. Pre‐Six‐month follow‐up: *p* < 0.05; RCI = N/A; Total in normal range = 74.8%.	Only included participants who completed the DP and therefore cannot identify drop‐out rates. Absence of family functioning related outcomes. Absence of control group.
Herpertz‐Dahlmann et al., 2014 **Program**: Five university hospitals and one major general hospital for general child and adolescent psychiatry. **Country**: Germany	Randomised controlled design; Cohort study.	**N** = 87 **Diagnostic groups**: AN = 100% **Gender**: All female **Age range**: 11–18 years *M* = 15.30 *SD* = 1.50	**Weight Measure**: BMI Estimated Body Weight (EBW) **Mental Health Measures**: Morgan and Russell Average Outcome and General Score (MRAOS; Morgan & Hayward,1988) EDI‐2 Brief Symptom Inventory (BSI; Franke, 2000) **Service Utilization Measure**: Number of readmissions for EDs	**Physical Health Outcomes**: Weight gain at discharge 80% of both day‐patient treatment and inpatient treatment, as measured by BMI. Gain of an average of 3 BMI units during treatment. No differences between day‐patient treatment and inpatient treatment at 12‐month follow‐up. Non‐inferiority of day‐treatment to inpatient treatment substantiated with similar results. *p* < 0.0001; 95% CI = −0.16 to 1.07	Sample included patients diagnosed with AN only. Limited site‐specific program description.
Hoste, 2015 **Program**: Comprehensive Eating Disorder Program at University of Michigan, Michigan **Country**: United States of America	Case series	**N** = 28 **Diagnostic groups**: AN = 71% OSFED = 29% **Gender**: Female = 90% Male = 10% **Age range**: 8–24 years *M* = 16.60 *SD* = 3.50	**Weight Measure**: EBW **Mental Health Measures**: Eating Disorder Examination‐Questionnaire (EDE‐Q; Fairburn & Beglin, 1994) CDI Centre for Epidemiological studies Depression Scale (CES‐D; Radloff, 1977) PvA	**Physical Health Outcomes**: Weight gain from admission to discharge for patients who completed the program. *p* < 0.001; *t* = ‒9.0 **Mental Health Outcomes:** Reduction in eating disorder symptomology, as measured by EDE‐Q subscales of Restraint, Eating Concern, and on Global Score. Pre‐post: *p* < .05; Average t‐score not reported. Increase in parent self‐efficacy for both mothers and fathers on PvA. p < 0.001, Average t‐score not reported. Reduction in depression symptoms for 18–24 year olds, as measured by CES‐D Pre‐post: p < 0.01	T‐scores not reported for self‐report measures. Absence of control, follow‐up, and drop‐out rates
Lazaro et al., 2011 **Program**: Eating Disorder Unit, Day Hospital Program, Barcelona. **Country**: Spain	Case series	**N** = 160 **Diagnostic groups**: AN = 59.4% BN = 17.5% EDNOS: 23.1% **Gender**: All female **Age range**: 13–18 years *M* = 15.50 *SD* = 1.20	**Weight Measure**: BMI **Mental Health Measures**: Piers–Harris Children’s Self‐Concept Scale (PHC‐SCS; Piers & Harris, 1969) Self‐Esteem in Eating Disorders Questionnaire (SEED; Trallero et al., 2005) Socialization Battery for Adolescents (BAS‐3; Silva & Martorell, 1989)	**Physical Health Outcomes**: Weight gain between start and end of treatment, as measured by BMI. Pre‐post‐ AN group = 0.9 points Pre‐post – BN group = 0.3 points **Mental Health Outcomes:** Gains in self‐concept as measured by PHC‐SCS. *p* < 0.05; *d* = −0.08 Gains in socialisation as measured by BAS‐3 subscales. Consideration for others *p* < 0.05; *t* = −1.80 Social withdrawal *p* < 0.05; *t* = 3.44	T‐scores not reported for changes in weight. Limited description of comorbidity. Limited generalisability of results as the study included those who responded moderately well to treatment. Absence of control, follow‐up, ED symptomology measures
Martin‐Wagar et al., 2019 **Program**: Adolescent day treatment program at a Midwestern eating disorder speciality clinic, Ohio. **Country**: United States of America	Case series	**N** = 87**Diagnostic groups**: AN–R = 71.26% AN–P = 28.73% **Gender**: Female = 92% Male = 8% **Age range**: 13–18 years *M* = 14.85 *SD* = 1.70	**Weight Measure**: BMI Expected Body Weight (EBW) **Mental Health Measures**: EDE‐Q Binge/purge frequency The Interpersonal Chaos subscale of the Life Problems Inventory (LPI; Rathus, Wagner, & Miller, 2005) The Family Empowerment Scale (FES; Koren, DeChillo, & Friesen, 1992)	**Physical Health Outcomes**: 66.7% of patients reached 95% EBW at discharge. 9.2% of patients reached 90‐94.5% EBW at discharge. 19.5% of patients reached less than 90%EBW at discharge. Unique predictors of weight restoration – Baseline BMI, four week %EBW weight gain, and baseline caregiver empowerment (as measured by FES) χ2 = 25.84; *p* < 0.001	Limited statistical testing comparing pre‐post on self‐report measures. Missing FES data at follow‐up. FES not commonly used as a measure of parental self‐efficacy (other studies using PvA) which makes it difficult to compare outcomes with other studies.
Ngo and Isserlin, 2014 **Program**: Eating disorder program at Children's Hospital, London, Ontario. **Country**: Canada	Case series	**N** = 49 **Diagnostic groups**: AN–R = 69.40% AN–P = 30.60% **Gender**: Female = 92% Male = 8% **Age range**: 13–17 years *M* = 15.30 *SD* = 1.20	**Weight Measure**: IBW	**Physical Health Outcomes**: Group IBW < 85% = 29 patients Mean %IBW at admission = 81.5 (2.5) Mean %IBW at discharge = 88.3 (5.4) Group IBW > 85% = 20 patients; Mean %IBW at admission = 88.0 (3.8) Mean %IBW at discharge = 92.2 (4.3)	No statistical analysis on weight gain between pre‐ to post‐treatment. Limited description of DP content. No self‐report measures utilise to assess mental health and family functioning.
Ornstein et. al, 2017 **Program**: Partial Hospital Program at Pennsylvania State Hershey Children's Hospital, Pennsylvania. **Country**: United States of America	Case series	**N** = 130 **Diagnostic groups**: AN = 52.30% ARFID = 24.6% BN = 11.5% OSFED/USFED = 11.5% **Gender**: Female = 92.3% Male = 7.7% **Age range**: 7–17 years *M* = 13.53 *SD* = 2.05	**Weight Measure**: MBMI **Mental Health Measures**: ChEAT Revised Children’s Manifest Anxiety Scale (RCMAS; Reynolds & Richmond, 1985).	**Physical Health Outcomes**: Weight gain across patients with ARFID, AN and OSFED/UFED. Patients with BN had significantly smaller change in %MBMI than patients with other EDs; *p* < .001; η^2^_p_ = 0.31 Patients showed significant improvement in weight, psychopathology over a short time period. **Mental Health Outcomes:** Gains in self‐concept as measured by PHC‐SCS. *p* < 0.05; *d* = −0.08	A retrospective chart review. Limited inferential statistics regarding changes in weight across diagnostic groups. Absence of control and follow‐up.
Ornstein et al., 2012 **Program:** Partial Hospital Program at Pennsylvania State Hershey Children's Hospital, Pennsylvania. **Country:** United States of America	Case series	**N** = 30 **Diagnostic groups**: AN = 52.30% BN = 11.5% EDNOS = 11.5% **Gender**: Female = 86.6% Male = 13.4% **Age range**: 8–16 years *M* = 12.80 *SD* = 2.00	**Weight Measure**: IBW BMI **Mental Health Measures**: ChEAT CDI RCMAS	**Physical Health Outcomes**: Weight gain between start and end of treatment, as measured by %IBW. *p* < 0.0001 At discharge from program, 87% of patients were at ≥90% IBW Weight gain between start and end of treatment, as measured by BMI. *p* < 0.0001 **Mental Health Outcomes:** Reduction in eating disorder symptoms as measured by ChEAT – total scores and all subscales. *p* < 0.001 Reduction in depression symptoms as measured by CDI – total score, and all subscales except ‘Interpersonal Problems’. *p* < 0.001 Reduction in anxiety symptoms as measured by RCMAS subscales: Total score *p* < 0.0001 Physiological anxiety *p* < 0.01 Worry / Oversensitivity *p* < 0.001 Social concerns / concentration *p* < 0.05	Limited description DP group interventions. Missing numerical data on ChEAT, CDI, RCMAS. Effect sizes not reported. Absence of control and follow‐up
Pennell et al., 2019 **Program**: Eating Disorder Program at the Regional Centre for Paediatric Eating Disorders, Ontario. **Country**: Canada	Case series	**N** = 24 **Diagnostic groups**: AN‐R = 41.6% AN‐B/P = 25% OSFED = 20.8% ARFID = 8% BN = 4% **Gender**: Female = 95.8% Male = 4.2% **Age range**: 13–17 years *M* = 15.42 *SD* = 1.25	**Weight Measure**: Body weight (kgs) **Mental Health Measures**: Frequency of binge and purge episodes upon discharge **Service Utilization Measure**: Readmission during the two‐year period.	**Physical Health Outcomes**: Weight gain between admission and discharge, as measured by body weight (kgs). *p* < 0.01; *t* = 4.87 Weight gain between admission and discharge, as measured by IBW. *p* < 0.01; *t* = 4.52 **Mental Health Outcomes**: Reduced frequency of binge and purge episodes at discharge for seven patients. **Service Utilization Outcomes**: Readmission reported for five patients.	Limited use of mental health measures assessing ED symptomology. Large number of missing data relating to frequency of binge‐purge episodes, and readmissions. No control group or follow‐up data.
Rienecke (2019) **Program**: Comprehensive Eating Disorder Program at University of Michigan, Michigan **Country**: United States of America	Case series	**N** = 87 Adolescent group (N = 55) Young adults group (*N* = 32): **Diagnostic groups**: AN = 100% **Gender**: Female = 90.8% Male = 9.2% **Age range**: Adolescent group: 10–17 years *M* = 14.10 *SD* = 1.70	**Weight Measure**: %EBW BMI **Mental Health Measures**: Mini International Neuropsychiatric Interview for Children and Adolescents (MINI‐Kid; Sheehan et al., 2010). EDE‐Q **Family Functioning Measure**: Family Questionnaire (FQ; Wiedemann et al., 2002).	**Physical Health Outcomes**: Higher %EBW in adolescent patients at the end of treatment, compared to those who dropped out of treatment. *p* < 0.05	No control group or follow‐up data. Limited analysis of change in %EBW from intake to discharge. Only AN patients included.
Rienecke & Richmond, 2018 **Program**: Comprehensive Eating Disorder Program at University of Michigan, Michigan **Country**: United States of America	Case series	**N** = 26 **Diagnostic groups**: AN = 76.9% EDNOS = 23.1% **Gender**: Female = 90.8% Male = 9.2% **Age range**: Adolescent group: 11–22 years *M* = 16.58 *SD* = 3.19	**Weight Measure**: %EBW **Mental Health Measures**: EDE‐Q CDI **Family Functioning Measure**: FQ PvA	**Physical Health Outcomes**: Higher %EBW in patients between admission and discharge. *p* < 0.01; *t* = ‐10.91 Higher %EBW in patients between discharge and three‐month follow‐up *p* < 0.01; *t* = ‐ 5.99 **Mental Health Outcomes**: Improvements in eating disorder symptoms between admission and discharge, as measured by EDE‐Q sub‐scales: Global Score *p* < 0.01; *t* = 5.89 Restraint *p* < 0.01; *t* = 5.74 Shape Concern *p* < 0.01; *t* = 3.40 Weight Concern *p* < 0.01; *t* = 4.93 Eating Concern *p* < 0.01; *t* = 6.29 Improvements in Weight Concern subscales of EDE‐Q, between discharge and 3‐month follow‐up: *p* < 0.01; *t* = 2.35 Reduction in depressive symptoms, as measured by CDI, from admission to discharge: *p* < 0.01, *t =* 2.92 Reduction in depressive symptoms, as measured by CES‐D, from admission to discharge: *p* < 0.01, *t =* 5.18 Increase in parent self‐efficacy found on the PvA for mothers, from admission to discharge: *p* < 0.01, *t =* −7.16 Increase in parent self‐efficacy found on the PvA for fathers, from admission to discharge: *p* < 0.01, *t =* −7.09 Decrease in parent self‐efficacy found on the PvA for fathers, from discharge to 3‐month follow‐up: *p* < 0.01, *t* = 2.98 Improvements in parental expressed emotions on the FQ, from admission to discharge, on the following sub‐scales: Critical comments *p* < 0.01, *t =* −5.04 Emotional over‐involvement *p* < 0.01, *t = ‐* 3.76 Improvements in parental expressed emotions on the FQ, from discharge to 3‐month follow‐up, on the following sub‐scale: Emotional over‐involvement *p* < 0.01, *t =* −2.97	No control group. Limited information on services / levels of care provided following discharge. Reliance on self‐report data for height and weight at 3‐month follow‐up.
Simic et al., 2018 **Program**: National and Specialist Child and Adolescent eating Disorder Service, South London, London. **Country**: United Kingdom	Case series	**N** = 105 **Diagnostic groups**: AN = 92.38% ARFID = 4.76% OSFED = 2.85% **Gender**: Female = 94.1% Male = 5.9% **Age range**: 11–18 years *M* = 15.50 *SD* = 1.50	**Weight Measure**: %mBMI BMI **Mental Health Measures**: EDE‐Q Mood and Feeling Questionnaire (MFQ; Costello & Angold, 1988) Penn State Worry Questionnaire‐Children (PSW‐C; Chorpita et al., 1997). Eating Disorders Quality of Life Scale (EDQLS; Adair et al., 2007) Motivational Ruler (MR; Miller & Rollnick, 2002) Intolerance of Uncertainty Scale (IUS; Buhr & Dugas, 2002) Negative Problem Orientation (NPO; D’Zurilla et al., 1999) Difficulties with Emotion Regulation Scale (DERS; Gratz & Roemer, 2004) Rosenberg Self‐Esteem Scale (RSES; Rosenberg, 1965)	**Physical Health Outcomes**: Weight gain at end of admission, as measured by %mBMI *p* < 0.001; *d* = 0.79 **Mental Health Outcomes**: Reduction in eating disorder symptoms, as measured by EDE‐Q *p* < 0.001; *d* = −0.85 Gains in eating disorder related quality of life, as measured by EDQLS. *p* < 0.001; *d* = 0.95 Gains in mood related symptoms, as measured by MFQ. *p* < 0.001; *d* = −0.89 Improvements in emotion regulation, as measured by DERS. *p* < 0.001; *d* = −0.52 Improvements in self‐reported confidence in ability to change, as measured by MR‐ability subscale. *p* < 0.001; *d* = 0.53 Gain in self‐esteem, as measured by RSES. *p* < 0.001; *d* = 0.45	No control group or follow‐up data. Majority of AN patients included. No inferential statistics for changes in BMI.
Smith et al., 2019 **Program**: Comprehensive Eating Disorder Program at University of Michigan, Michigan **Country**: United States of America	Case series	**N** = 51 **Diagnostic groups**: AN‐R = 70.6% AN‐A = 21.6% AN‐BP = 7.8% **Gender**: Female = 94.1% Male = 5.9% **Age range**: 9–17 years *M* = 14.10 *SD* = 1.70	**Weight Measure**: %EBW BMI **Mental Health Measures**: MASC Eating Disorder Examination Interview (EDE; Cooper & Fairburn, 1987) Subjective Units of Distress Scale (SUDS; Wolpe, 1990)	**Physical Health Outcomes**: Weight gain at end of admission, as measured by %EBW *p* < 0.01; *t* = −12.78 Reduction in meal‐related anxiety, as measured by SUDS. *p* < 0.01; *t* = 4.09	No control group or follow‐up data. Only AN patients included. Difference scores utilised for analysis of SUDS scores.
Zanna et al., 2021 **Program**: Anorexia Nervosa and Eating Disorder Unit, Bambino Gesu Children's Hospital, Pediatric Unit, Rome. **Country**: Italy	Pilot Study	**N** = 34 **Diagnostic groups**: AN = 93.84% EDNOS = 6.16% **Gender**: All female **Age range**: 11–18 years *M* = 15.28 *SD* = N/A	**Weight Measure**: Percentiles of body mass index (%BMI) **Physical Health Measure**: Heart rate **Mental Health Measure**s: MASC‐2 CDI‐2 Achenbach Youth Self‐Report (YSR) (Achenbach & Rescorla, 2007) EDI‐3 Body Uneasiness Test (BUT) (Cuzzolaro, Vetrone, Marano, & Batacchi, 1999) MROAS **Family Functioning Measure**: Family Assessment Device (FAD) (Epstein, Baldwin, Bishop, 1983) **Service Utilization Measure**: Cost assessment using the Health Care Financing Administration‐Diagnosis Related Group (HCFADRG) system, Version 24.	**Physical Health Outcomes**: Weight gain between start and end of treatment in day program as measured by %BMI. *p* < 0.001; *r* = 0.60 Higher effect size values for %BMI in day program group compared to inpatient group – with increase in %BMI with a large effect size only detected in day program group. *p* < 0.001; *r* = 0.54 **Mental Health Outcomes:** Reduction in symptoms in patients in day program (compared to inpatient unit) as measured by MROAS subscales Eating difficulties *p* < 0.0001; *r* = 0.73 Mental state *p* < 0.01; *r* = 0.43 Insight *p* < 0.0001; *r* = 0.63 Intimate relationships *p* < 0.001; *r* = 0.52 Social contacts *p* < 0.001; *r* = 0.50 Occupation *p* < 0.0001; *r* = 0.68 **Hospital Costs:** No difference found in health care costs between day program and inpatient care. *p* = 0.54; *r* = 0.11	Majority of included patients with AN. Non‐randomised clinical trial. Small sample size (n = 17 in each group), which may be the reason for no differences between IP and HLCT in terms of clinical and psychological parameters at pre‐treatment. No post‐treatment mental health measures utilised. Short treatment period. No follow‐up data

#### 2.4.6 Staffing

Seventeen studies reviewed reported to have multi‐disciplinary teams (MDT) while four studies (Bustin et al., 2013; deGraft‐Johnson et al., 2013; Rienecke, 2019; Smith et al., 2019) did not make a specific reference to an MDT. Nine studies included a pediatrician (Ornstein et al., 2012; Grewal et al., 2014; Henderson et al., 2014; Ngo & Isserlin, 2014; Hoste, 2015; Simic et al., 2018; Smith et al., 2019; Pennell et al., 2019; Martin‐Wagar et al., 2019). Ten studies mentioned including nursing staff. DPs in eighteen reviewed studies reported including a psychiatrist. Fourteen studies reported including a dietitian. Lastly, eight studies included teachers as part of the educational support provided to patients in the DPs (deGraft‐Johnson et al., 2013; Girz et al., 2013; Grewal et al., 2014; Henderson et al., 2014; Hoste, 2015; Ornstein et al., 2017; Pennell et al., 2019; Rienecke, 2019).

#### 2.4.7 Program structure

DPs typically operated for seven hours per day ranging from 5 (Goldstein et al., 2011) to 10 h a day (Henderson et al., 2014). Four studies did not report the daily duration of DPs (Bustin et al., 2013; Girz et al., 2013; Grewal et al., 2014; Herpertz‐Dahlmann et al., 2014). Of those five studies which reported on details of operating hours, one operated in the afternoon from 2pm to 8:30pm (Lazaro et al., 2011), while the other four operated from the morning to late afternoon (e.g., 8 AM to 4 PM; deGraft‐Johnson et al., 2013; Ngo & Isserlin, 2014; Simic et al., 2018; Pennell et al., 2019).

ED‐DPs operated between three and half days per week (Goldstein et al., 2011) to 5 days per week (Lazaro et al., 2011; Ornstein et al., 2012; deGraft‐Johnson et al., 2013; Grewal et al., 2014; Henderson et al., 2014; Hoste, 2015; Ornstein et al., 2017; Bryson et al., 2018; Simic et al., 2018; Rienecke & Richmond, 2018; Pennell et al., 2019; Martin‐Wagar et al., 2019; Rienecke, 2019; Smith et al., 2019). Eight studies determined the duration of the DP on an individual basis depending on weight gain and maintenance (Lazaro et al., 2011; Ornstein et al., 2012; Bustin et al., 2013; Girz et al., 2013; Gewal et al., 2014; Simic et al., 2018; Pennell et al., 2019; Martin‐Wagar et al., 2019). Five studies did not report proposed DP duration (Herpertz‐Dahlmann et al., 2014; Ngo & Isserlin, 2014; Hoste, 2015; Ornstein et al., 2017; Bryson et al., 2018). Five studies (Goldstein et al., 2011; deGraft‐Johnson et al., 2013; Henderson et al., 2014; Rienecke, 2019; Smith et al., 2019) reported treatment episode lengths to be 7 weeks on average, ranging from 2.6 weeks (deGraft‐Johnson et al., 2013) to 12–14 weeks (Henderson et al., 2014).

#### 2.4.8 Interventions and activities

DPs in most studies included the following programs: individual patient psychotherapy session, patient group counselling, individual parent counselling, single family counselling with a patient and a parent, parents group counselling, multi‐family group counselling, at least two supervised meals and nutritional counselling. Variations were noted in how the DPs implemented intervention components. For example, most DPs required patients' parents to attend at least one supervised meal a day (e.g., Hoste, 2015; Smith et al., 2019) while one program allowed for patients to have staff supervised meals without the presence of parents (Martin‐Wagar et al., 2019).

Of the studies reviewed, one study included motivational enhancement strategies (Goldstein et al., 2011) and sibling psychoeducation session (Goldstein et al., 2011). One study utilised phone coaching between therapist and parents which involved discussing appropriate strategies for parents to provide meal support for their children over the weekend (Girz et al., 2013). Parents were required to attend the DP facility during the weekdays to provide support if patients refused to finish a meal (Girz et al., 2013). Two studies included art therapy (Goldstein et al., 2011; Ornstein et al., 2017) and one study included yoga (Henderson et al., 2014). Six studies reported that their DPs had school‐based activities incorporated into their daily schedule (deGraft‐Johnson et al., 2013; Henderson et al., 2014; Ngo & Isserlin, 2014; Ornstein et al., 2017; Pennell et al., 2019; Rienecke, 2019). Three studies indicated running daily school‐based activities for 2 to 2.5 h for 5 days per week (deGraft‐Johnson et al., 2013; Ornstein et al., 2017; Pennell et al., 2019).

All the studies utilized FBT with or without other additions or adaptations. Eleven studies used FBT‐informed care. Six studies implemented FBT and incorporated CBT‐E approaches as part of the treatment (Goldstein et al., 2011; Lazaro et al., 2011; Herpertz‐Dahlmann et al., 2014; Ngo & Isserlin, 2014; Ornstein et al., 2017; Bryson et al., 2018). One study used both FBT and DBT‐informed treatment approaches together (Pennell et al., 2019). Three studies utilized an FBT‐informed care, CBT and DBT‐informed approach (Simic et al., 2018; Martin‐Wagar et al., 2019; Smith et al., 2019).

#### 2.4.9 Number of Admissions

Most studies (*n* = 17) did not report the maximum capacity of DP. Three studies reported to have a capacity of up to eight people (Girz et al., 2013; Grewal et al., 2014; Ngo & Isserlin, 2014). Simic et al. (2018) reported providing care for eight to ten patients per day and Pennell et al.’s (2019) DP provided care for four to six patients per day.

#### 2.4.10 Drop‐out rate

Drop‐out is generally defined as discontinuing treatment prior to the recommendations of the treating service. According nine of the reviewed studies which reported drop‐out rates, the average drop‐out rate was 21.38%. Three studies classed patients who were admitted to IP while undertaking DP as drop‐out (Goldstein et al., 2011; Ornstein et al., 2017; Simic et al., 2018). Two studies considered patients who discontinued the DP due to ED‐related medical condition as drop‐out (Bryson et al., 2018; Ornstein et al., 2017). Four studies did not specify the detailed criteria for drop‐out despite reporting drop‐out rate (Grewal et al., 2014; Herpertz‐Dahlmann et al., 2014; Hoste, 2015; Martin‐Wagar et al., 2019).

#### 2.4.11 Parental involvement

All reviewed studies required parent participation. Six studies specified that their DPs required at least one parent to participate in the program interventions (Lazaro et al., 2011; Ornstein et al., 2012; Girz et al., 2013; Rienecke & Richmond, 2018; Rienecke, 2019; Smith et al., 2019). Nine studies specified that at least one of their supervised meals involved a parent (Ornstein et al., 2012; Girz et al., 2013, Henderson et al., 2014; Hoste, 2015; Ornstein et al., 2017; Simic et al., 2018; Pennell et al., 2019; Martin‐Wagar et al., 2019; Smith et al., 2019). The remaining six studies did not specify the nature of parent involvement.

#### 2.4.12 Physical health outcomes

All reviewed articles included weight measures assessed by a medical practitioner. All the studies reported an increase in weight by the end of the admission to the DP. Four studies did not conduct inferential statistical analyses to assess changes in weight (Girz et al., 2013; Grewal et al., 2014; Lazaro et al., 2011; Ngo & Isserlin, 2014), with several studies not reporting on effect sizes. Of the studies that reported effect sizes related to changes in body weight post‐treatment, two studies reported small effect (*d* = 0.30 to 0.50; Goldstein et al., 2011; *r =* 0.10 to 0.30; Bryson et al., 2018) and two studies reported medium to large effect sizes (*d* = 0.50 and higher; Simic et al., 2018; *r =* 0.30 and higher; Bryson et al., 2018; Zanna et al., 2021). Goldstein et al. (2011) reported medium to large effect sizes in six‐month follow‐up (*d* = 0.86).

Seven studies reported on follow‐up outcomes. Six studies reported that patients maintained their weight gain at 3, 6, 12 or 31 months post discharge. Only Zanna et al. (2021) discussed using the measurement of any other metabolic monitoring indicator (heart rate).

It is important to note that there were variations in how body weight was measured and reported. Most (*n* = 18) included one measure and few studies (*n* =3) included two measures. Of all reviewed studies, six studies calculated ideal body weight (IBW), and seven studies assessed changes in weight by BMI. Three studies calculated ideal body weight based on MBMI and five studies determined the deviation from ideal body weight by Expected Body Weight (EBW) (Herpertz‐Dahlmann et al., 2014; Hoste, 2015; Martin‐Wagar et al., 2019; Rienecke, 2019; Smith et al., 2019).

EBW can be calculated in three different ways: BMI percentile, McLaren method, or Moore method (Le Grange et al., 2012). The BMI percentile method involves using the 50^th^ BMI percentile as the patient’s goal weight – based on their age and gender (Le Grange et al., 2012). Of five studies which used EBW to track weight recovery, one studies utilised the BMI percentile method (Herpertz‐Dahlmann et al., 2014). The Moore method estimates ideal body weight according to height percentile on a growth chart based on age (Moore et al., 1985). For instance, a 12‐year‐old patient with a height in the tenth percentile, should be at the tenth percentile for weight for 12‐year‐old population norms (Moore et al., 1985). Three studies utilised the Moore method (Rienecke & Richmond, 2018; Martin‐Wagar et al., 2019; Smith et al., 2019). Finally, the McLaren method determines ideal body weight by drawing a vertical line between the child's height on the height‐for age graph and the equivalent 50th percentile weight (McLaren & Read, 1972). None of the studies utilised the McLaren method. One study was unclear about which method was used to calculate EBW (Rienecke, 2019)

It is important to note that there are moderate correlations amongst these three methods. The more discrepant calculations were seen at high and low height percentiles and in older teens (Kanget, et al., 2019; Le Grange et al., 2012). Lastly, one study calculated Goal Weight (GW) to monitor weight gain without specifying the formula for GW calculation (Grewal et al., 2014).

#### 2.4.13 Psychological outcomes

Most of the studies utilized self‐report measures only (*n* = 15). Eleven studies used one measure and four studies used two measures to assess ED‐related outcomes. Six studies did not measure any ED‐related outcomes. Measures that were utilized across at least two studies are discussed below.

#### 2.4.14 Eating disorder inventory 2nd edition

EDI‐2 is a valid 91‐item self‐report questionnaire used to evaluate behavioural and psychological psychopathology of EDs in patients aged over 12 years. Herpertz‐Dahlmann et al. (2014) compared EDI‐2‐Global Score between a DP and an IP at pre‐treatment and 12 months follow‐up and found no significant difference, indicating IP service was not superior to DP. EDI‐2‐Body Dissatisfaction showed gradual reduction between pre‐treatment to 6 months follow‐up. However, only EDI‐2‐Body Dissatisfaction between pre‐treatment to 6 months follow‐up was statistically significant, showing approximately 80% of patients returned to healthy range.

#### 2.4.15 Eating disorder inventory 3rd edition

EDI‐3 is a 91‐item self‐report inventory used to assess psychological traits and behavioural symptoms associated with AN, BN, and EDNOS in those aged between 13 to 53 years. Two DPs used EDI‐3 and reported significant improvement in all subscales administered except for EDI‐3‐Maturity Fears at post‐treatment. Goldstein et al. (2011) administered five subscales (Drive for Thinness, Body Dissatisfaction, Perfectionism, Personal Alienation, Maturity Fears) of EDI‐3 in those with AN and EDNOS subtype AN. They found significant reduction in Drive for Thinness and Perfectionism, with a small to medium effect sizes post‐treatment (*d* = 0.11 to 0.58) and medium to large effect sizes at 6 months follow‐up (*d* = 0.49 to 0.99). Body Dissatisfaction scale showed statistically significant reduction between pre to 6 months follow‐up only, but not between pre‐ to post‐treatment (Goldstein et al., 2011). Girz et al. (2013) administered all subscales of EDI‐3 in those with AN, BN, EDNOS‐subtype AN and EDNOS‐subtype BN. They found significant reduction in all subscales except for EDI‐3‐Maturity Fears between pre‐ to post‐treatment, which was consistent with Goldstein et al. (2011). Finally, Baudinet et al. (2020) found changes across several EDI‐3 subscales (Table 2) with small to medium effect sizes, ranging from *d* = 0.26 to 0.39. The biggest effect sizes were reported for the subscales of Personal Alienation and Interceptive Deficits (Baudinet et al., 2020). Long‐term outcome of EDI‐3 was not assessed in any of the studies reviewed.

#### 2.4.16 Children's eating attitudes test

ChEAT is a valid 26‐item self‐report questionnaire that was developed to assess eating attitudes and behaviors associated with AN and BN (Garner, Olmsted, Bohr, & Garfinkel, 1982; Maloney, McGuire & Daniels, 1988). All four studies which used ChEAT reported improvement for eating attitudes and behaviors in ED patients (Ornstein et al., 2012; Ornstein et al., 2017; Bustin et al., 2013; Bryson et al., 2018).

Ornstein et al. (2017) compared ChEAT‐total and all three subscales amongst patients with AN, BN, ARFID and OSFED/UFED at pre‐ and post‐treatment. As expected, they found that both AN and BN scored high and within clinical range on ChEAT‐total while those with ARFID scored in the subclinical range on ChEAT‐total at pre‐treatment. At discharge, no differences amongst four diagnostic groups were found on ChEAT‐total score, and those with AN and BN experienced a significantly greater reduction relative to those with ARFID. Similarly, Bustin et al. (2013) found a reduction on ChEAT total scores following day program treatment. Consistent with Ornstein’s (2017) findings, Bryson et al. (2018) found that those with AN scored significantly higher on ChEAT‐total compared to those with ARFID. Both AN and ARFID's ChEAT‐total scores decreased at a similar rate from post‐treatment to 31 months follow‐up (Bryson et al., 2018).

#### 2.4.17 Eating disorder examination questionnaire

EDE‐Q is a 36 item self‐report questionnaire used to evaluate behavioural and cognitive features of EDs (Fairburn and Beglin, 1994). Three studies found significant improvement in EDE‐Q total score at post‐treatment (Hoste, 2015; Simic et al., 2018; Rienecke & Richmond, 2018). Hoste (2015) further identified that both Restraint and Eating Concern subscales showed significant improvement at post‐treatment while Shape Concern and Weight Concern subscales did not. Simic et al. (2018) reported a large effect size at post‐treatment for changes in EDE‐Q scores (*d* = −0.85). Rienecke & Richmond (2018) found significant improvements on all EDE‐Q sub‐scales between admission and discharge (subscale *t‐*scores ranging between 4.93 and 6.29; see Table 2). The authors found significant gains between discharge and three‐month follow‐up for the Shape Concern subscale (see Table 2; Rienecke & Richmond, 2018).

#### 2.4.18 Other psychological measures

All reviewed studies utilized self‐report measures to assess depression and anxiety symptoms. Three studies used one measure and four studies used two types of measures. Measures that were utilized across at least two studies were Children’s Depression Inventory (CDI; Kovacs, 2010; *n* = 6), Revised Children’s Manifest Anxiety Scale (RCMAS; Reynolds & Richmond, 1985; *n* = 2), Morgan and Russel Average Outcome Score (MRAOS; Morgan & Hayward, 1988; *n* = 3) and the Multidimensional Anxiety Scale for Children (MASC; March, 1997; *n* = 2). Of the six studies that utilized the CDI, four reported significant reductions in depressive symptoms at discharge (Girz et al., 2013; Henderson et al., 2014; Ornstein et al., 2012; Rienecke & Richmond, 2018). Similarly, the improvements in anxiety and general functioning were observed in studies utilizing the MRAOS, RCMAS and MASC at discharge (Henderson et al., 2014; Ornstein et al., 2012; Zanna et al., 2021). Regarding follow‐up data, Girz et al. (2013) found significant reductions in depressive and anxiety symptoms at three‐ and six‐month follow‐up utilizing the CDI and MASC respectively. Overall, these findings highlight gains in comorbid mental health concerns following ED‐DP admissions.

## 3 DISCUSSION

The present review aimed to examine whether DPs for children and adolescents with EDs were efficacious in treating ED symptoms and promoting physical and psychological health. In the studies reviewed, children and adolescents admitted to ED‐DPs showed significant improvement in eating disordered symptoms. The effect sizes for weight gain post‐treatment ranged from small to large. There is some evidence to also support the maintenance of these gains in studies that conducted 6‐month (Goldstein et al., 2011) and 31‐month follow‐up of the patients (Bryson et al., 2018). Of note, Goldstein et al. (2011) reported large effect sizes related to weight gain at 6 months post‐treatment. The varying findings across the studies can be attributed to differences in measurements and program evaluation methodology.

The review highlights several variations in how body weight was measured and reported between the programs and studies. For instance, changes in physical health were assessed using various measures of body weight. Commonly used weight indices included BMI, IBW, EBW, and MBMI. Several clinical practice guidelines for the treatment of EDs (e.g., Hay et al., 2014) have no specific recommendations for indices of body weight despite its importance in tracking recovery (Lebow et al., 2018). While differences in measurement in the studies reviewed reflects practices within DPs, variations in the calculation of weight gain have been observed across studies of ED in children and adolescents in general (Le Grange et al., 2012). Consensus around best practice in such measurements may help benchmark ED‐DP outcomes around the world.

Similar to the measurement of physical health concerns, there was variance between programs about how psychological health gains were measured. Amongst the studies that utilised psychological measures, commonly used measures of ED symptoms included the EDI‐2, EDI‐3, ChEAT, EDE‐Q, and mood and anxiety was assessed by CDI, RCMAS and/or MASC. All of these measures were well‐validated and held good reliability amongst child and adolescent populations. With many of the ED treatments focusing on changes within the family, the utilisation of measures of parent and family functioning would be useful. For example, programs utilising FBT‐based approach would benefit from more consistent use of parent‐rated measures, such as the Parents versus Anorexia Scale (e.g., Hoste, 2015; Rienecke & Richmond, 2018). Measures of general functioning (e.g., Children's Global Assessment Scale), functional impairments (e.g., Adaptive Behaviour Assessment System) and wellbeing and quality of life (e.g., Perceived Quality of Life) may also be of benefit. Overall, there needs to be more consistent use of reliable and valid measures of ED symptoms to enable comparisons to be made between studies.

Seven studies did not report drop‐out rate and those studies that reported drop‐out differed in their definition of drop‐out. For instance, some studies considered patients who did not complete DP due to inpatient transfer as dropouts and one study categorised those who did not achieve weight restoration as dropouts (Grewal et al., 2014). This is concerning as it biases study results toward stronger efficacy of DP. Therefore, there needs to be more consistency in reporting of drop‐out rate and criteria.

The common elements identified were DPs operating five days a week, the use of FBT‐informed care, utilizing multi‐disciplinary teams, having some measure of weight and using of self‐report measures. Apart from these commonalities, significant variations and omissions were noted in the description and details provided regarding program elements in the papers reviewed. Four articles included detailed descriptions of the services. As the DPs reviewed in this paper are from around the world, peer‐reviewed journal articles on the program evaluations of these DPs may consider a description of the services and the health care service context to support the generalisation of learning to the international community of paediatric ED health professionals. All reviewed DPs employed MDT staff, however, there was minimal information about the training and minimum requirements for MDT staff to be eligible to work at ED‐DPs. Also, there was little clarity of the role and contributions of specific disciplines in the ED interventions with children and adolescents with various EDs.

Most studies reported that their DP typically ran for seven hours per day for 5 days per week. However, several studies did not report their program operating hours and duration. Operating hours and duration are an important consideration when thinking about the feasibility and cost‐effectiveness of programs. For example, it may be beneficial to investigate whether clients engaging in DPs with a shortened duration of time also report similar benefits. If so, such models may be more feasible in health care contexts with limited resources. This may be the case for DPs in countries that lack the mental health care infrastructure that is readily available to the programs reviewed in the study. Operating hours of the DP also have implications in terms of the burden and level of disruption it is to a child and family’s life. More time at a DP relates to having more time away from ones' home and school, and possibly more time away from work and other family members for parents. This has implications for the child or adolescent reintegrating into home, school and other relevant contexts following discharge. Most DPs appear to be linked to a hospital. Given ED‐DP is part of a stepped care model, being located within a hospital system would enable clients to receive intensive, inpatient services in a timely manner if required. However, it remains unclear if the transition to community service from such hospital‐based services is more difficult for children, adolescents and their families.

All studies utilised an FBT‐informed care approach with some including other evidence‐based interventions such as CBT‐E and DBT. There was a wide variation in the types of additional interventions and activities (e.g., art therapy, yoga, nutritional counselling; individual parent counselling, parents group counselling). With several of these interventions utilised currently lacking an evidence base to validate their efficacy, there is a need for ED‐DPs to more rigorously evaluate the efficacy of these novel interventions with children, adolescents and families. It would be useful to identify any unique contributions of these alternate approaches, over and above those gained through evidence‐based treatments like FBT‐informed care or CBT‐E. In addition, as supervised meals provide a parent with an opportunity to learn skills to support their child’s eating behaviours, it may be useful to report the degree of parental involvement (e.g., preparing meal, supporting a child to eat) in supervised meal session. Lastly, information about the number of inpatient admissions during an ED‐DP admission was also inconsistently reported. Information about the use of nasogastric feeding during DP admissions may also be of benefit in program evaluations.

## 4 FUTURE DIRECTIONS

With research now pointing to the multi‐organ impact of EDs, ED‐DPs should report on other physical health indicators – including heart rate and blood pressures (Hay et al., 2014; Sachs et al., 2016; Tokumura et al., 2012). While patients to the ED‐DPs are typically admitted based on the criteria of being ‘medically stable’, the periodic physical health assessment may alert health professionals to any comorbid health conditions that require attention (Vo et al., 2016).

The studies reviewed did not consistently report if participants were prescribed psychiatric medication whilst at ED‐DPs. There is much debate on the use of psychopharmacology in EDs with children and adolescents (Flament et al., 2012) – with some emerging evidence to suggest that the use of second‐generation antipsychotics (SGA) may be of benefit to adolescents with comorbid mental health concerns (Baeza et al., 2017; Flament et al., 2012; Leggero et al., 2010). With inconsistent reporting of the use of medications and medication adherence, it is unclear how much of the reported psychological benefits of the program can be linked to the use of medication.

Most studies reported requiring clients to be medically stable upon ‘stepping down’ from inpatient service to ED‐DP, however many did not specify admission criteria for clients who 'stepped up' from outpatient service to ED‐DP. At present, there is little consensus about how decisions about moving clients across the continuum of ED services are made (Butterfly Foundation, 2015). The development of research evidence to inform criteria used for admission and discharge from ED‐DPs may build a clearer rationale for the use of such services. While specific expertise relating to FBT and other specific intervention approaches may be important, there is evidence to suggest that recovery‐oriented interventions – aimed at improving overall functioning rather than just focus on the remission of mental health symptoms – may be of benefit for clients (Dawson et al., 2014). An understanding of how professionals from different disciplines contribute to such recovery processes may help build the rationale for the need for an MDT in ED‐DPs.

## 5 CONCLUSION

The present review concluded, based on the best available research, that ED‐DPs for children and adolescents with EDs are beneficial in promoting physical and psychological recovery. It remains unclear which components of the DPs contribute to recovery. Variations exist between the outcomes or components of ED‐DPs and these can be attributed to the differing clinical and demographic profiles of the clients, issues related to the existing mental health service infrastructure within the country in which the DP was situated, the inclusion of novel interventions, and lack of evidence‐based practices with ED‐DPs. Innovations in the development of mental health services requires further evaluation and validation of the use of a continuum of care and greater consistency in the description of DP models and the children and adolescents accessing these services. The review highlights and supports the use of evidence‐based treatments, such as FBT‐informed care and CBT‐E within ED‐DPs. With increasing scrutiny over the use of health care resources, and the need for validated, evidence‐based practices, robust evaluation and reporting of such mental health services may help with advocating for the early intervention of conditions like EDs in children and adolescents.

6

## Data Availability

Data sharing is not applicable to this article as no new data were created or analyzed in this study.
